# Methodological reflections: developing the WHO database of violence against women policies

**DOI:** 10.1093/heapol/czad052

**Published:** 2023-08-09

**Authors:** Katherine Watson, Eva Burke, Judy Gold, Gillian Eva, Avni Amin

**Affiliations:** Freelance Consultants; Freelance Consultants; Freelance Consultants; Freelance Consultants; Department of Sexual and Reproductive Health and Research, World Health Organization, 20 Avenue Appia, 1211 Geneve, Switzerland

**Keywords:** Violence against women, health policy, gender, policy analysis

## Abstract

This paper presents methodological reflections from the development of the World Health Organization (WHO) Violence against Women (VAW) Policies Database (hereinafter referred to as ‘the Database’) to inform future efforts to create similar public health policy databases for government accountability. Using the WHO Global Plan of Action on Violence accountability measures as a starting point, the Database was developed over a 2-year period in consultation with a reference group. A subset of indicators was piloted before finalization of a full list and the structure of the Database. Available VAW policies from 194 WHO Member States were reviewed by a team of consultants, who conducted content analysis and data entry. A ‘Manual and User Guide’ was developed to record decisions related to the processes for developing the Database. This guide was used to draw out key reflections in relation to policy indicators, inclusion criteria for policy documents, languages and analysis, quality assurance and sustainability. The process of developing the Database evolved iteratively in response to many factors, including the content of policies and the specificities of policy-making in each jurisdiction. Pragmatic decisions about the number of indicators and the types of policies to review were informed by pilot tests across a range of geographies. Standardization of analysis and data entry was ensured through the provision of in-depth guidance for researchers, and regular and open communication within the team was key to quality assurance. Online translation services enabled a review of policy documents in most languages. Documentation of the methodology ensured that others could replicate processes with fidelity in the future. Despite complexities, it is possible to develop a sound methodology for analysing the content of policy documents in a manner that yields findings that are useful in holding governments accountable for the commitments to address VAW and other public health issues in policy.

Key messages

- Given the diversity in how countries reflect issues within policy documents, precise sub-indicators improve reliability in interpreting and categorizing policy data. The number of indicators used should be kept to a reasonable number to ensure that the policy monitoring exercise is manageable over time.
- Securing up-to-date policy documents is an onerous task, and those that are likely to contain the most relevant content should be prioritized. Pragmatic decisions need to be made as to which policies should be prioritized for inclusion within the boundaries of available time and resources.
- Given the variability of terminology used across regions, ensure that search terms are translated by native speakers and address cultural sensitivities. To reduce bias, in-depth guidance should be developed for researchers that aids in the consistent interpretation of policy content.
- Quality assurance measures are invaluable in ensuring standardization and should be built into policy analysis process. While very useful, double entry of data is time intensive; as such, there is need for flexibility and innovation in the quality assurance methods used.
- Sustainability of a policy database is reliant on the availability of resources for future updates, which requires that there is upfront investment in the database architecture to ‘future proof’ it in anticipation of changes over time. It is important that methodologies are well documented so that anyone who is tasked with updates is able to replicate the process with reasonable fidelity.

## Introduction

Violence against women (VAW) and girls is not only a major human rights violation rooted in gender inequality but also a public health problem that has devastating consequences on health and well-being. The World Health Organization (WHO) estimates show that, globally, almost one in three women (30%) aged ≥15 years has experienced physical and/or sexual violence from a male intimate partner and/or sexual violence from someone other than an intimate partner at least once in their lifetime since the age of 15 (World Health Organization, 2021a). In 2016, WHO Member States endorsed resolution WHA69.5—the *Global Plan of Action to Strengthen the Role of the Health System within a National Multisectoral Response to Address Interpersonal Violence, in particular Against Women and Girls, and Against Children* (‘the Global Plan of Action on Violence’) at the World Health Assembly (World Health Assembly, 2014), which specifies policy options for health and multisectoral responses to VAW.

Preventing and responding to VAW requires a range of responses and interventions with individuals, families, communities and institutions across multiple sectors including police, justice, education, social protection and health. Policies are one component of a comprehensive, multi-pronged, multi-level approach to preventing and responding to VAW. When implemented with the backing of adequate human, financial and technical resources and in partnership with communities, however, policies serve as an important element of a comprehensive approach to addressing VAW. With this in mind, in 2020, the WHO commissioned the development of a database to house information on the content of VAW health and multisectoral policies from around the world. The database—known as the VAW Policies Database (hereinafter referred to as ‘the Database’)—aimed to establish a baseline against which to monitor progress against the Global Plan of Action on Violence, facilitate policy dialogues with Member States^1^ and support countries to align their national health and multisectoral policies with WHO recommendations, international human rights standards and other evidence-based strategies to address VAW (World Health Organization, 2021b).

The need for and the difficulty of comparative policy analysis has been well documented by those working across a range of health, equity and well-being issues, with many authors highlighting the tensions that exist in such exercises. Efforts to analyse policy in relation to individual topics have a myriad of limitations, including the number of countries, comparability of measures and comprehensiveness (Marmor *et al.*, 2005). The developers of the Global Abortion Policies Database, e.g. highlight that policy interpretation is very contextual, which poses a challenge for policy researchers tasked with analysing legal and policy texts across contexts (Johnson *et al.,* 2018). Others have noted the tensions that exist between (1) the long-term nature of the policy cycle and the short-term nature of funding for policy research and (ii) the depth of information that can be systematically obtained and the number of countries to be included in a comparative analysis (Walt *et al.*, 2008; Raub *et al.,* 2022). Despite these challenges, all authors agree that assessing policies is a crucial starting point for improving health outcomes.

## Overview of the database development process

A team of researchers and WHO staff members purposefully and meticulously documented the Database development process through the elaboration of a manual and user guide that was continuously updated to reflect the team’s methodological decision-making. The development of the Database took place across three phases. First, Pilot phase during which indicators were developed, refined and tested. Next, during Phase 1, data for a subset of 12 indicators were populated for all 194 countries. Analysis of findings from Phase 1 and reflections on the data entry processes led to further refinements ahead of Phase 2, during which data for 54 indicators were populated in the Database for all countries.

The starting point for developing indicators was the accountability measures defined in the Global Plan of Action on Violence. The indicators were developed iteratively, eventually covering six domains of measurement: enabling policy environment, guiding principles of woman-centred care, provision and availability of health services for survivors, inclusion of interventions for populations living in vulnerable situations, prevention strategies and availability of VAW prevalence data. Information for each of the indicators was populated in the Database based on the content analysis of countries’ policy documents.

**Figure 1.**
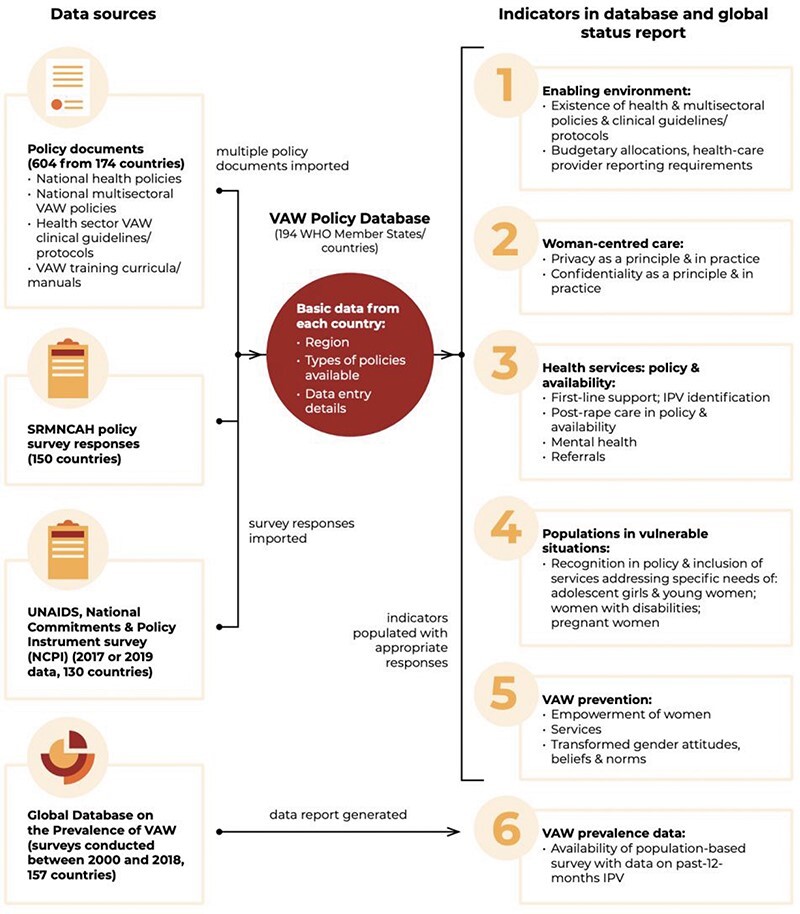
Overview of the VAW Policies Database development from WHO (2021b)

Relevant policy documents for each country were identified in a number of ways. First, a repository of policy documents collated from countries’ responses to the WHO 2018–2019 Sexual, Reproductive, Maternal, Newborn, Child and Adolescent (SRMNCAH) Policy Survey yielded policy documents from 155 countries (World Health Organization, 2020). Additional documents were obtained through targeted online searches, WHO regional offices repositories and outreach to country offices requesting the most up-to-date policy documents. Two existing datasets were also used to populate some of the indicators: (1) the National Commitments and Policy Index survey administered by the Joint United Nations Programme on HIV/AIDS (UNAIDS) that included data on availability of post-rape care and (2) the WHO Global Database on the Prevalence of VAW (UNAIDS, No date; World Health Organization, No date) (see Figure 1 for further detail on data sources).

Four types of policy documents were included for content analysis: national health policies, health sector guidelines/clinical VAW protocols, national multisectoral VAW action plans and health worker training curricula related to VAW. To standardize content analysis of these policy documents conducted by a team of eight researchers, search terms were developed in English and translated into three other United Nations (UN) languages—Spanish, French and Arabic. The team of researchers were either native or bilingual speakers of these four UN languages. For the rest of the UN languages and for non-UN languages, two online translation platforms (Google Translate and DeepL) were used to translate the documents into English. Once content was extracted using the search terms and interpreted according to the standardized guidance, data relevant to each indicator were entered in a bespoke, online platform developed for the project. Aggregated data reports were generated by the Database and used to present global data and regionally disaggregated data for the six WHO regions.

A reference group was constituted by the WHO to advise on the development of the Database. The group was composed of external experts in the area of policy research, policymakers, civil society and WHO regional offices and other UN partners, including those with experience developing policy databases. The advice of the reference group was critical in gaining a better understanding of diverse policy development contexts, including details of how and when policies related to health and violence against women are developed, updated, used and given weight by governments over time. The group provided advice at three key moments—namely, before the completion of each of the project’s aforementioned phases. During the Pilot phase, the reference group members’ recommendations and guidance included: to expand the inclusion criteria to include expired policies in the absence of a more up-to-date policies for each country; to include an indicator on referral services from the health sector to other sectors and to create separate indicators for psychosocial support and mental health care for survivors. They also provided insights on response categories, search strategies, policy inclusion criteria and data entry during the pilot and subsequent phases.

This paper describes five aspects of the methods in more detail with reflections about the specific challenges, choices and lessons learned to share with policy researchers in global health. This includes (1) the development of indicators; (2) inclusion criteria for policy documents; (3) languages and standardizing content analysis; (4) quality assurance processes and (5) creating a sustainable database architecture.

## Development of the indicators

At the outset of the project, the intention was to develop a database around countries’ responses to the 2017–2018 WHO SRMNCAH policy survey (World Health Organization, 2020). However, given that the surveys were not necessarily completed by respondents familiar with VAW policies in each country and given the absence of information on the specific policy documents upon which survey responses were based, a decision was made to develop a new set of indicators that could be populated from the primary analysis of policy documents.

Fifty-nine indicators and response categories (or answer options) were developed during the Pilot phase for internal testing across 10 geographically diverse countries. While the testing affirmed that information on most of the indicators could be found in policies, it also highlighted the diversity of policy content therein. The Pilot phase findings were shared with the reference group, which affirmed the thematic focus of indicators and advised a reduction in the number of indicators. There were some minor alterations to the wording of various indicators following the Pilot phase but no other substantive changes.

Prioritizing fewer indicators for Phase 1 ensured that data would be available in time for the World Health Assembly’s 5-year progress review on the Global Plan of Action on Violence in May 2021. During this phase, data for 12 indicators were populated into the Database for all 194 countries. In addition to indicators that related to the existence of policies and budget for VAW, the indicators chosen for this phase related to whether the following were found in the available policies for each country:

1. Commitment to train health providers on VAW;
2. Auditory/spatial privacy during consultation with survivors;
3. Confidentiality of survivors’ information;
4. First-line support for survivors (i.e. psychological support);
5. Mental health referral for survivors;
6. Mental health assessment for survivors;
7. Differentiated services for adolescent girls and young women survivors and
8. Gender norm change interventions for preventing VAW.

Similar to the findings of the Pilot phase, reflections on and findings from Phase 1 highlighted that policy content varied significantly for certain indicators. However, with a much larger dataset to analyse, these variations emerged more clearly and, crucially, highlighted inconsistencies in the application of response categories for certain indicators. For example, analysis of the ways in which privacy was reflected in policies’ content showed that there were two main variations: (1) privacy was mentioned as a guiding principle of all service provision and (2) privacy was applied in the context of consultations with survivors, often with more practical guidance for service providers. Guidance in Phase 1 did not necessarily assist researchers in determining which of these ‘variations’ fully or partially met the criteria for the privacy indicator.

Box 1. Refining indicators: example of an indicator related to inclusion of right to privacy in policy

**Table:** 

Indicator	Main response categories
PILOT + PHASE 1	
Are the (following) components of woman-centred health care included in health policy? (privacy during consultation)	Yes, fully included Yes, partially included Not specified
PHASE 2	
Aggregate indicator based on two sub-indicators: Does policy include the privacy component of woman-centred care?	Yes, privacy as principle included Yes, privacy in consultations included Yes, privacy in principle and in consultations included Not specified
Sub indicator 1: Does policy include privacy as a principle of woman-centred care? Sub indicator 2: Does policy include respect for privacy during consultation?	Yes, included Not specified

As a result of these Phase 1 findings, more precise sub-indicators were developed to measure specific components. For example, the indicator on inclusion of privacy was split into one aggregate indicator and two sub-indicators that reflected the above-mentioned variations (Box 1). The aggregate indicators were computed based on the response categories for sub-indicators. This approach not only increased reliability but also provided more nuanced insights into the ways that countries address the right to privacy in their policy guidance. The pattern of splitting broad indicators into sub-indicators was replicated in relation to indicators covering the right to confidentiality, the inclusion of populations living in vulnerable situations and the prevention of VAW.

Splitting out indicators into more precise sub-indicators contributed to an expansion in the total number of indicators from 59 in the pilot phase and Phase 1 to 74 at the outset of Phase 2. The research team, together with the reference group and the WHO, prioritized the long-list of 74 indicators based on those for which existing WHO guidelines or human rights standards existed as well as the expressed evidence needs of countries; 54 indicators were chosen for Phase 2.

Key learnings

- Policy indicator development is an iterative process. It requires piloting and refinement with clear and precise definitions and response categories that are informed by stakeholders involved in policy-making and familiar with how policies are developed at the country level.
- Given the diversity in how countries reflect issues within policy documents, more precise sub-indicators can improve reliability and allow for more precise and standardized interpretation and categorization of qualitative or text-based policy data.
- While it can be challenging to prioritize, the number of indicators needs to be kept to a reasonable number to ensure that the policy monitoring exercise is manageable over time, while also having sufficient indicators to ensure the breadth of information required. Referencing existing evidence and considering the expressed evidence needs of those involved in policy-making are good ways to narrow down lists of indicators.

## Inclusion criteria for types of policy documents

As with the development of indicators, the inclusion criteria for policy documents evolved over time. During the Pilot phase, a range of policy documents available from the SRMNCAH Policy Survey repository were accessed and reviewed; these included national health and multisectoral policies and strategies and protocols relating to VAW, human immodeficiency virus (HIV), sexual and reproductive health (SRH), maternal health, adolescent health and mental health. The research team conducted targeted searches within all of these types of policies to determine the likelihood of finding VAW content in each. Weighing this up alongside considerations of the time and resources available, decisions had to be made about which types of policies to include and exclude for data entry.

Six types of policy documents were included for review in Phase 1: (1) national health policies relating to general health, HIV and SRH only; (2) national multisectoral VAW plans; (3) national health sector/clinical protocols to respond to VAW; (4) health worker training manuals and curricula to guide VAW response; (5) national VAW monitoring and evaluation frameworks and (6) ‘other,’ for documents with relevant information not falling into the first five categories. Following Phase 1 data entry, however, it became clear that Types (5) and (6) were rarely available and, if they were, they did not have useful additional content on VAW prevention and response. These two types of policy documents were excluded in Phase 2, and information from them that was already populated into the Database during Phase 1 was removed.

At the same time, Phase 1 highlighted the importance of including reproductive, maternal, newborn, child and adolescent (RMNCAH) national health policies, given the frequency with which these were turning up in searches for VAW policies online and the fact that many countries had RMNCAH policies as opposed to SRH policies. The definition of what constituted a ‘national multisectoral VAW plan’ was also expanded to include national plans related to gender equality. RMNCAH national policies and gender equality policies were thus added for Phase 2.

Although the original plan was to include a number of other additional types of policy documents in Phase 2, including adolescent, mental and maternal health policies, this was not possible due to feasibility concerns. The number of policy documents referenced in the Database during Phase 1 exceeded 600, and the research team felt that it would not be feasible to search additional types of policy for the expanded list of 54 indicators within the given timeframe. Similarly, a decision was made to exclude sub-national policies, even though, in many federated countries, policies are made at the sub-national level. These were excluded given that sub-national policies are hard to find online and require time and effort to obtain.

Key learnings

- A policy database that relies on content analysis of policy documents needs to have a plan for obtaining up-to-date policy documents. It is an onerous task to obtain these manually, and efforts need to be made to procure these through various sources including through outreach to countries, online searches and country surveys.
- To make it feasible to analyse vast amounts of content contained in the policy documents requires prioritizing those that are most likely to contain content relevant to VAW prevention and response. This can be a challenge, as content on VAW prevention and response and particularly in relation to health was found to be dispersed across different topics and types of documents.
- Given the varying degrees of detailed content in policy documents, there is a need to triangulate across different levels and types. For example, a multisectoral plan of action on VAW may include high-level details of the health sector’s response, which is then expanded upon in health sector–specific policy documents.

## Languages and standardizing content analysis

For content analysis of the policy documents, search terms were developed in English and translated by native speakers familiar with VAW terminologies into Spanish, French and Arabic. In many cases, the terms could not be directly translated and had to be adapted to region-specific terms. For example, the terminology for intimate partner violence has a great deal of variation across regions, with policies referring to ‘domestic violence’, ‘family violence’ or ‘spousal violence’; all such terms were included in searches. In addition, due to cultural sensitivities associated with direct translation of the term ‘intimate partner violence,’ a more suitable term in Arabic (‘Eunf elSharik’) was used.

To minimize researcher bias in the interpretation of policy content, a ‘data entry dashboard’ was developed with definitions of each indicator and guidance. The definitions and guidance included a range of different search terms that could be synonyms or illustrate the content or concept in different ways across different countries. Updating the data entry dashboard continually allowed for improvements to be made to the process in real time, but it also presented challenges in ensuring that all team members kept up with and implemented those changes. Regardless of attempts to standardize, it is important to acknowledge the unavoidable subjectivity of individual researchers and, also, the limitations of interpreting policy documents without in-depth knowledge of each country’s context.

Key learnings

- Given the variability of terminology used across regions, it is important to ensure that search terms are translated by native speakers and address cultural sensitivities. Challenges related to working with English as the default language from which all terms and standards are translated highlight the need to adopt a multi-lingual approach to development of search terms and concepts.
- To reduce bias, in-depth guidance for researchers—in the form of a ‘data entry dashboard’ or other, similar tool—that aids in the consistent interpretation of policy content is crucial for quality and standardization. This requires elaborating on different terms, synonyms and concepts that speak to a particular indicator or meet the indicator requirements across different policy documents and languages.

## Quality assurance processes

Quality assurance processes were built into every phase of the Database’s development in order to minimize errors and maximize consistency in interpretation of the content in the policies across the different team members doing the data entry. For example, during the Pilot phase, the research team undertook double entry of data for 10 countries; in Phase 1, double entry was completed for the first 3 countries assigned to new team members and in Phase 2, no double entry was possible due to the timeline. Double entry allowed two individuals to independently look at the same policy document and enter their interpretation as a response to the indicator. Where the same text was interpreted differently by the two researchers, it allowed for discussions to improve interpretation of the text in a standardized way.

Another example of a quality assurance process was the use of a ‘buddy system’ whereby each team member was paired with someone else on the team, who acted as a sounding board for questions and clarifications. This, too, allowed for a standardized approach to content analysis and categorization of responses. When differences in interpretation or questions persisted between the ‘buddies’, they were elevated to the research team lead to offer clarification or a solution. In both phases, a designated quality assurance team member conducted ‘spot checks’ on data entered for randomly selected countries to ensure consistency.

During Phase 2, a mid-term analysis of four indicators was conducted to identify inconsistencies and update guidance for indicators that the research team found challenging. The four indicators chosen for analysis were first-line support, mental health, populations in vulnerable situations and transformation of norms. For each, all entered data were scanned by the team lead and the designated quality assurance team member to determine whether the policy content extracted for analysis was accurately classified in the response category. For example, the analysis found that there were a high number of countries, for which the ‘not specified’ response category had been used for the mental health treatment indicator. Following this, clarifications on what type of text or terms that qualified as ‘mental health treatment’ were made. Findings from the mid-term analysis for all four indicators were presented to all team members, and the written guidance in the data entry dashboard was updated. Each team member was required to review and, if needed, update the response categories for each of their assigned countries in the Database and employ the new guidance for their remaining countries.

Key learnings

- Quality assurance measures are invaluable in ensuring standardization when working to quantify qualitative information contained as text that is articulated differently across settings.
- There are several approaches to assure quality of the data entry that should be built into the policy analysis process. This includes double entry of all or a sample of countries’ documents, a buddy system and spot checks of data entered, along with regularly updated explanations of content or text that meet the requirements of the indicator.
- Quality assurance processes are time and labour intensive. For example, double entry of data for 54 indicators for all 194 WHO countries required more time and resources than were available. With limited time and resources, researchers can adopt flexible, innovative methods for quality assurance that evolve with the needs of the database.

## Creating a sustainable database architecture

Currently, the Database provides a snapshot of the policy framework for each country at a certain point in time—i.e. as of November 2021. The Database does not as yet have the functionality to enter data from new(er) policies without replacing the existing data. Therefore, it does not allow tracking the evolution of policies within a country over time. Adding this functionality to the Database would be valuable for sustainability and for monitoring the commitments under the Global Plan of Action on Violence over time. While the Database is established to track four types of policy documents, there is scope to expand this and include additional types of policy documents. Likewise, new indicators that emerge as being important for tracking government accountability can be added in the future. Adding new features to ensure the continued value and use of the Database by intended audiences also requires feedback from end users.

A key challenge relates to the human and financial resources required to update the contents of the Database reasonably frequently (e.g. at least once every 3–5 years). Future updates will require trained researchers to update data in a manner that is consistent with the initial investment. To partially enable this, the Database is accompanied by a detailed ‘data dictionary’, data entry dashboard and user guide.

Key learnings

- Sustainability of a policy database is reliant on the availability of resources for future updates, which requires that there is upfront investment in the database architecture to ‘future proof’ it in anticipation of changes over time.
- Within the scope of available resources, incremental improvements to the Database can be made over time to improve sustainability and require a concerted effort to reach out to end users to obtain their feedback.
- It is important that methodologies are well documented so that anyone tasked with updating the Database will be able to replicate the process with reasonable fidelity.

## Reflections

In reflecting on a complex undertaking of creating a VAW Policies Database, key learnings emerged that are relevant for policy researchers seeking to undertake similar exercises either in relation to VAW or any other public health policies. Based on these learnings, the following reflections are offered in relation to indicator development, policy inclusion criteria, languages and standardizing analysis, quality assurance and sustainable database architecture.

First, in developing standardized policy indicators, an iterative process is usually required, recognizing that countries develop policies in a variety of ways across multiple sectors. An initial set of indicators can be developed collaboratively with relevant stakeholders, including those working on developing national policies, and draw upon existing evidence. Indicators can be piloted and re-piloted several times for fine-tuning. It is generally not possible to capture every detail or nuance of policies without making the exercise overwhelming. On the one hand, there should be a reasonable number of precise policy indicators that provide critical information about how policies address VAW or any other area of health. On the other hand, steps should be taken to ensure that the data to be collected do not exceed the time and resources available for the project or generate a volume of data that will not be useful to audiences developing policies or holding governments accountable to those policies. Achieving this balance will materialize differently for every policy database.

Second, there will always be scope to broaden out the types of policies included in policy research; as such, starting with well-defined inclusion criteria and a small set of policy document types is advised so long as inherent limitations are clearly articulated. If needed, the types of policy documents included can later be expanded out according to the availability of human and financial resources. Existing repositories of policy documents are good starting points and, while online searches do yield a wealth of additional documents, the ideal scenario is to do direct outreach to policymakers in each context to ensure that the policies being reviewed are the most up-to-date and relevant to the exercise.

Third, to ensure equity and inclusion, every effort should be made to include policies in a variety of languages, including non-UN languages. If language capacities are not available within a given research team, online translation platforms are a practical alternative. Utilization of these platforms substantially expands the range of documents and countries included—and at a much lower cost than requiring full review by a fluent speaker of each language. This is particularly important for some regions—such as Europe. These translation platforms have limitations that must be recognized, including requiring particular document formats (e.g. they often will not accept scanned documents) and translating from some languages more accurately than others. Online translation platforms are not recommended for the translation of search terms; rather, native speakers with familiarity of the subject matter should be involved in setting and refining them so that culturally sensitive terminologies are used rather than direct translations.

Fourth, recognizing the subjectivity in interpreting qualitative content in policy documents that is written in a variety of ways, it is important to provide standardized definitions and guidance. A ‘data entry dashboard’ or similar tool can be developed that provides definitions of each indicator, key search terms in all languages and tips or advice derived from real-time review of data entered to date. This type of tool can be developed iteratively throughout the research process, with updates being communicated with all researchers as and when they are made.

Fifth, quality assurance mechanisms should be built intentionally in order to improve reliability of the data. While double entry is a ‘gold standard’, other methods—including some of those listed in this paper—can be used and adapted where resources are limited. Having a team member with dedicated time for implementing quality assurance measures is a useful and pragmatic approach to ensure that this is prioritized throughout data entry.

Finally, it should be acknowledged that undertaking a global policy review requiring an associated database to manage is a time-, human- and financial resource-intensive undertaking. While not always possible to envision all its future uses, consideration should be given at the outset to whether a database will remain static or be continually updated and, if so, by whom, how and with what frequency. With the VAW Policy Database, it was not clear at the outset if and how it would be used and updated beyond the initial data entry and analysis, and thus, the database build did not factor in these requirements. Databases that will be updated regularly require a different design from the outset, and decisions such as whether old data will be archived or replaced must be made.

Documentation of other policy databases has drawn out good practices for overcoming the limitations of constructing globally comparative indicators on a variety of topics related to health, equity and well-being. The WORLD Policy Analysis Center’s documentation of the development of the parental leave policy database highlights the importance of (1) referencing primary data sources, (2) defining essential features of policy with reference to existing research and global treaties, (3) developing coding frameworks that capture the richness of approaches across contexts, (4) reviewing a sample of countries’ policies to develop categories, (5) developing a coding manual for researchers and (6) working with a coding team that is fluent in multiple languages and can apply the coding framework consistently across countries, among other factors (Raub *et al.*, 2022). For the VAW Policies Database, the team was able to apply these same recommendations in full, reinforcing their good practices as relevant for another area of health policy research. In addition, our team has been able to contribute further, detailed reflections in relation to the iterative processes involved in indicator development, policy selection, research standardization and quality assurance, while also sharing experiences using online translation platforms for policy review.

It is crucial for database development to evolve iteratively in order to encompass the richness and diversity of health policy across contexts and to respond to the multitude of known and unknown variables that arise. Despite the inherent challenges, the Database’s development was the first step towards understanding countries’ commitments and intentions for preventing and responding to VAW. Going forward, the findings will be used to hold governments accountable for VAW policies that are grounded in evidence as well as gender equality and human rights standards.

## Data Availability

No new data were generated or analysed in support of this research.

## Footnotes

1. Only countries that are WHO Member States were included in the Database. Hereinafter, the term ‘countries’ is used in place of ‘WHO Member States.’

## Reflexivity statement

The authors agree with and acknowledge the importance of having authors from low and middle-income countries as an important part of decolonizing global health efforts. Please note that this particular policy research was commissioned by the WHO as a global exercise that was intended to reflect variations at the regional level. While this first phase of analysis did not involve researchers from LMIC institutions, ongoing plans to update the Database and conduct further research are inclusive of WHO and Ministry of Health colleagues across all regions and Member States. Furthermore, in 2023 and beyond, there are plans to engage researchers and policymakers from LMICs to develop case studies to assess the ‘policy readiness’ of the health system in relation to VAW policy.

All five authors of the paper identify as women and are located in the Americas, Europe, Asia and Australia, respectively. After a competitive bidding process, a team was selected, and four of the authors of this paper are members of that team who led/contributed to the methodological development of the Database. The lead from the WHO side – Dr Avni Amin – although currently based in Geneva, Switzerland, is an Indian national and has spent a significant chunk of her personal life in India and of her professional life working with Ministries of Health in LMICs.

## References

[R1] Johnson BR Jr , Lavelanet AF, Schlitt S. 2018. Global Abortion Policies Database: a new approach to strengthening knowledge on laws, policies, and human rights standards. *BMC International Health and Human Rights* 18: 35. 10.1186/s12914-018-0174-2 PMC6134502 30208877

[R2] Marmor T , Freeman R, Okma K. 2005. Comparative perspectives and policy learning in the world of health care. *Journal of Comparative Policy Analysis: Research and Practice* 7: 331–48.

[R3] Raub A , Sprague A, Waisath W et al. 2022. Utilizing a comparative policy resource from the WORLD policy analysis center covering constitutional rights, laws, and policies across 193 countries for outcome analysis, monitoring, and accountability. *Journal of Comparative Policy Analysis: Research and Practice* 24: 313–28.

[R4] UNAIDS . No date. Laws and Policies: National Commitments and Policy Index. https://lawsandpolicies.unaids.org/about?lan=en, accessed 22 May 2023.

[R5] Walt G , Shiffman J, Schneider H et al. 2008. ‘Doing’ health policy analysis: methodological and conceptual reflections and challenges. *Health Policy and Planning* 23: 308–17. 18701552 10.1093/heapol/czn024 PMC2515406

[R6] World Health Assembly . 2014. Resolution 67.15. Strengthening the Role of the Health System in Addressing Violence, in Particular Against Women and Girls, and Against Children. https://apps.who.int/iris/handle/10665/162855, accessed 22 May 2023.

[R7] World Health Organization . 2020. Sexual, Reproductive, Maternal, Newborn, Child and Adolescent Health Policy Survey, 2018–2019: Report. https://apps.who.int/iris/handle/10665/331847, accessed 22 May 2023.

[R8] World Health Organization . 2021a. Violence Against Women Prevalence Estimates, 2018. https://www.who.int/publications/i/item/9789240022256, accessed 12 December 2022, Last accessed: 22 May 2023.

[R9] World Health Organization . 2021b. Addressing Violence Against Women in Health and Multisectoral Policies: A Global Status Report. https://www.who.int/publications/i/item/9789240040458, accessed 22 May 2023.

[R10] World Health Organization . No date. Global Database on the Prevalence of Violence against Women. https://srhr.org/vaw-data, accessed 22 May 2023.

